# Autism and the Environment?

**Published:** 2005-06

Autism spectrum disorders (ASD) are a group of neurodevelopmental disorders that emerge before 3 years of age and are characterized by impairments in social and communicative skills and the presence of stereotyped and repetitive behaviors and interests. The prevalence of ASD appears to have increased dramatically within the last decade. Intensive community-based surveys estimate that as many as 6 of 1,000 school-age children are affected. Although part of the increase can be attributed to changes in diagnosis and greater public awareness, there is concern that increased exposure to toxic environmental agents during critical periods of brain development may play a role.

Much of the existing data used to implicate environmental agents in ASD is limited by methodological shortcomings and has not addressed the issue of gene–environment interactions. In recognition of the public health importance of understanding autism and the lack of reliable data that bear on potential environmental etiologies, the NIEHS has taken steps to support research in this area. The largest effort has been through the NIEHS/EPA Centers for Children’s Environmental Health and Disease Prevention Research (**http://www.niehs.nih.gov/translat/children/children.htm**), where two centers focus on autism.

These centers, located at the University of California, Davis, and the University of Medicine and Dentistry of New Jersey, are conducting multidisciplinary studies to identify environmental and genetic risk factors in autism. Strong partnerships have been formed between community advocacy groups and center investigators and have been used to develop and refine the studies to be conducted. Ongoing projects include epidemiologic and clinical investigations of risk factors and the development of animal and cellular models to examine the interaction of candidate neurotoxicants with signaling pathways and molecules that have been implicated in autism.

In addition to these centers, the NIEHS works collaboratively with other NIH institutes to support broader autism initiatives and activities. As one example, Program Announcement (PA) 04-085, Research on Autism and Autism Spectrum Disorders (**http://grants.nih.gov/grants/guide/pa-files/PA-04-085.html**) describes the broad scope of autism research topics of interest to the NIH. The NIEHS encourages applications with a primary focus on environmental factors that may influence autism risk or phenotypic expression. Other topics may be suitable for support from other participating institutes.

## Contact

**Cindy Lawler, PhD** | lawler@niehs.nih.gov

## Figures and Tables

**Figure:**
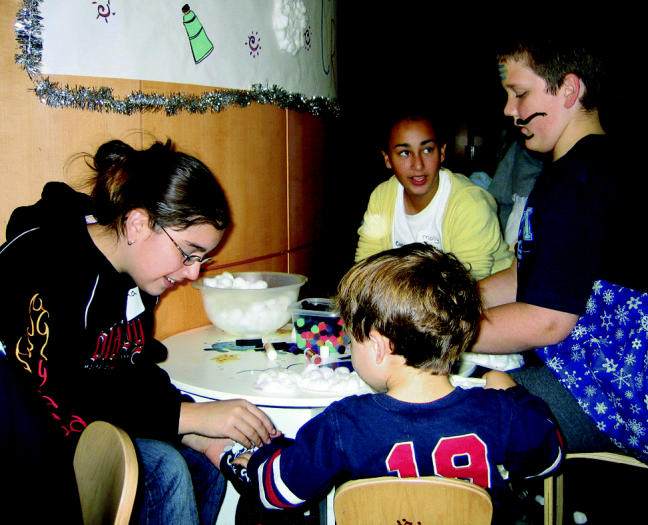
Participants and their families at UC Davis M.I.N.D. Institute annual holiday party.

